# Influence Mechanism of Teacher Support and Parent Support on the Academic Achievement of Secondary Vocational Students

**DOI:** 10.3389/fpsyg.2022.863740

**Published:** 2022-04-18

**Authors:** Xianjie Peng, Xiaonan Sun, Zhen He

**Affiliations:** ^1^Institute of Vocational Education, Tongji University, Shanghai, China; ^2^Faculty of Education, Beijing Normal University, Beijing, China

**Keywords:** parent support, teacher support, academic performance, secondary vocational school students, self-determination theory

## Abstract

Teachers and parents are key participants in the growth of secondary vocational students, and can jointly support them. This study aimed to explore how the support of teachers and parents affects the academic performance of secondary vocational students and to reveal the ‘black box’ mechanism for their interaction and relationship. We adapted a Chinese version of a scale for secondary vocational students’ perception of teacher and parent support, drawing on self-determination theory. A survey was conducted through the Chinese questionnaire platform *wjx.cn*, and respondents were fully informed about the research. Data were collected from 710 secondary vocational students in Shanghai and analyzed using SPSS and AMOS. Our hypothesis model was verified, with results indicating that the support of autonomy, emotion, and ability provided by parents and teachers has a significant impact on students’ academic performance. The degree of learning engagement plays a mediating role in the relationship between support and achievement. The achievement goal orientation of students can adjust individual learning engagement, and further, affect the influence of teacher and parent support on academic performance. Teacher and parent support interactively influence learning engagement. The study findings suggest that an optimization strategy is needed to promote the academic improvement of secondary vocational students to meet students’ basic psychological needs and promote cooperation between family and school. Parents and teachers should also consider the impact of learning engagement and the learning process, and offer guidance in developing an appropriate achievement goal orientation and a positive learning concept.

## Introduction

Teacher and parent support are important resources that can significantly impact the development of students’ academic performance (Zimet et al., 1988). Teacher support is a direct factor in the development of student academic achievement (Glozah and Pevalin, 2014), and students’ perception of teacher support stimulates autonomous learning motivation and interest in learning (Deci and Ryan, 2000). The family also provides an important environment for the growth of students, and family education is a foundation and supplement for school education (Fan, 2021). Many studies have confirmed that equal and reciprocal parent–child relationships result in support for children in terms of autonomy, emotion, and ability. Through parent support, children can cultivate their ability to solve problems independently and improve academic performance (Qingyu et al., 2010). However, for many years, researchers have paid more attention to students in ordinary high schools than those in secondary vocational schools. Further research is needed into the influence mechanism of teacher and parent support on the academic performance of secondary vocational school students.

In the Chinese context, academic performance is still regarded as an important indicator of students’ learning quality. Academic results influence learning and the quality of vocational education development in China (Yongsheng and Lejun, 2019). The current Chinese secondary school entrance examination system results in relatively poor academic performance of secondary vocational students, and such students are often considered to have limited potential. In turn, these negative perceptions have led to obstacles in the development of vocational education in China (Guirong et al., 2016). However, students can always improve when they are provided with support. Secondary vocational students are usually eager to improve their abilities and seek recognition and respect, which are important emotional supports needed for their further development (Jie, 2021). These students are the reserve force of junior and intermediate skilled personnel in the labor market (Guirong and Jiajia, 2020).

Schools and families are important for students’ development, and the supportive behaviors of teachers and parents can help students form positive responses and achieve pleasing academic results (Farkas and Grolnick, 2010). Teachers can provide support through encouragement, treating students fairly, and providing them with development opportunities. Students’ perception of the support, in turn, has a positive impact on their academic performance (Brophy and Good, 1969). Learning engagement is an attitude to learning and a key indicator of students’ emotions toward learning and their degree of participation (Marks, 2000). It has a continuous impact on students’ academic performance and is affected by external factors such as schools and families (Heping et al., 2020). The relationship between learning engagement and academic performance is the best predictor of students’ academic performance (Shernoff and Hoogstra, 2001), and students with high levels of learning engagement in schools achieve better academic results (Skinner and Belmont, 1993). In contrast, teachers’ behavior is the most critical factor affecting learning engagement, and improving teachers’ behavior is an effective way of enhancing students’ learning engagement (Haerens et al., 2013).

Achievement goal orientation reflects students’ motivations and rationales for engaging in achievement-related behaviors and has an important impact on academic performance (Pekrun et al., 2009). It includes three categories: mastery goal orientation, achievement-approaching goal orientation, and achievement-avoiding goal orientation (Harackiewicz et al., 1998). To improve performance, parents can help their children by providing support in terms of autonomy, emotion, and ability (Zong et al., 2017). In families, students’ educational expectations are influenced by parents, leading to different levels of learning engagement (Guiqing and Yang, 2018). Parents may share their time, financial resources, knowledge, and skills to help improve their child’s learning engagement, ultimately benefitting their academic performance (Green et al., 2007).

This study focused on secondary vocational school students in Shanghai in the context of the development of secondary vocational education in China. General academic performance of secondary vocational students was used as an objective indicator to measure the quality of learning, and five questions were posed and addressed from the perspective of self-determination theory: What type of support do teachers and parents use to influence the academic performance of secondary vocational school students? What is the interactive path and relationship between support and academic performance? What variables play a mediating or regulating role in the relationship between support and academic performance? What is the internal influence mechanism? How does revealing the internal mechanism help us improve the academic performance of secondary vocational students?

## Theoretical Framework and Research Hypothesis

Self-determination theory was proposed by Deci and Ryan in the 1980s and has evolved into a theoretical system of self-evaluation covering cognitive appraisal theory, organic integration theory, causal orientation theory, basic psychological needs theory, and goal content theory. The present study focused on the influence of the external environment on individual academic performance, and self-determination theory was considered appropriate for several reasons. First, individuals depend on their own self-determination ability and whether they can make a free choice regarding their own behavior under the premise of fully understanding their own needs (Zhanguang and Xiangkui, 2005). Second, the theory considers that the individual pursuit of self-determination constitutes behavioral motivation. It regards motivation as a multidimensional concept that can be divided into three categories: intrinsic motivation, extrinsic motivation, and amotivation (Jian et al., 2011). Third, the type of behavioral motivation is influenced by an individual’s perception of the external environment, which is divided into two categories: the sense of autonomy and the sense of being controlled. At the same time, satisfaction with autonomy helps to enhance students’ intrinsic motivation (Xianglan, 2017). Fourth, if the external environment can satisfy an individual’s sense of competence, ability, and belonging, it will help stimulate their innate internalization and integration tendency, transforming the external rules and requirements into the value of inner identity (Vansteenkiste et al., 2006). Therefore, this theory is applicable when explaining the influence of parent and teacher support on the development of secondary vocational students’ academic performance.

Students’ academic performance is an important indicator of the quality of their learning and can be used to evaluate the learning effect. The relationship between students’ academic performance and social support is a theoretical and practical issue of concern. Social support refers to the assistance provided through an individual’s social relationships. A benign social support atmosphere can stimulate students’ learning motivation and produce positive learning behaviors that enhance academic achievementı (Zheng et al., 2015). Teacher and parent support are important social supports for students’ development.

Studies have shown that students who perceive more social support from important people in their environment make greater gains in their academic performance than students who do not (Rosenfeld et al., 2000). Social support theory considers that an individual’s organized interpersonal network can provide expressive and instrumental support for individual development (Cullen, 2006). Expressive support refers to an individual’s sharing of emotions with others who are regarded as significant, gaining recognition of their value and dignity from others, and obtaining advice and guidance to meet their needs (Sumner et al., 2015). Instrumental support refers to material support from others, such as financial help provided by parents (Brouwers and Tomic, 2016). Functionally, social support that affects students’ academic performance mainly refers to the material, financial and spiritual support provided by significant others such as parents and teachers. Practically, the expressive and instrumental support from significant others needs to be fulfilled through social network relationships (Sarason et al., 1990). An optimal social atmosphere brought about by students’ social network relationships will stimulate students’ learning motivation, produce positive learning behaviors, and improve academic performance (Zheng et al., 2015). Although all social support impacts students’ academic performance, parents and teachers play the greatest role in children’s and adolescents’ physical and mental development and academic performance because of their authoritative role. Therefore, this study explores the influence mechanism of teacher and parent support on the academic performance of secondary vocational school students.

Parents and teachers play the role of protectors in students’ development, and the supports of autonomy, emotion, and ability provided by them have an important impact on students’ academic performance (Hualing et al., 2020). In the context of self-determination theory, parent support refers to the degree of support students perceive from their parents for their decisions. Parent support is reflected in the degree to which students obtain useful information from their parents and the emotional recognition they receive (Nishimura et al., 2021). Secondary vocational students are developing autonomy and independence, and are typically eager for activities outside the family. Parent support in this stage often manifests in providing emotional and instrumental support, helping form a strong psychological connection between child and parents that plays an important role in alleviating the pressures faced and promoting healthy growth (Laible et al., 2000).

Teacher support refers to the help and perception of autonomy, emotion, and ability that students acquire through teachers (Xianyun and Shaoying, 2013). A series of comparative studies showed that students who perceive more teacher support perform significantly better academically than those who perceive less teacher support (Wei and Yingying, 2018). Students with a greater perception of teacher support have stronger learning motivation, leading to better academic performance. In contrast, students who perceive less teacher support tend to focus on avoiding criticism, which negatively impacts learning effectiveness and academic performance (Deci and Ryan, 2000).

Social support has an interactive effect on student development, and parent and teacher support usually jointly influences students’ academic performance. Studies have divided parent–teacher interactive support into three categories: the enhancement mode means that one type of support can enhance the impact of another kind. For example, students who establish a supportive relationship with their parents are more likely to establish a supportive relationship with their teachers, and the two relationships will enhance each other to optimize the student’s development (Hongyv, 2012). Compensation mode is when one type of support is deficient or counterproductive, leading the other support to compensate for its negative impact. For example, when a student perceives less autonomy support from teachers, parent autonomy support will play a greater role in their academic development (Qin et al., 2013). Independent mode refers to sources of support and their independent effects on individuals. For example, studies have found that parent and teacher support have independent effects on individuals, and their relative importance depends on individual self-evaluation (Hongyv, 2012). On the basis of this analysis, we proposed the following hypotheses.

H1: Parent support and teacher support have an independent significant impact on the academic performance of secondary vocational school students.

H2: Parent support and teacher support have an interactive effect on the academic performance of secondary vocational school students.

Further research is needed to clarify the role of learning engagement in the influence mechanism of parent and teacher support on the academic performance of secondary vocational school students. Learning engagement refers to students’ attitudes about learning and is an important indicator of their learning effect. It is a multidimensional concept that includes enthusiasm, the emotional experience related to learning activities, and the cognitive strategies used in the learning process (Fredricks et al., 2004). Learning engagement has a sustained positive impact on students’ academic performance and future development. First, the external environment (e.g., family and school) and internal personality factors (e.g., learners’ achievement goal orientation) will affect students’ learning engagement. In terms of the external environment, school and family are the main places for learning activities. The higher the level of parent and teacher support perceived by students, the more interest they will have in learning and the more time and energy they will devote to it (Wei and Yingying, 2018). Teachers’ attention to students’ social development and psychological needs in school, characteristics of class assignments (Helme and Clarke, 2001), and classroom management behavior (Rongrong, 2019) will significantly impact students’ learning engagement. Parental occupation, education level, income level, educational expectations of children, and the level of educational involvement will affect the students’ learning engagement (Leishan et al., 2013). Second, the time and energy that students devote to educational activities is a strong predictor of development. Students who experience pride and satisfaction in the process of learning tend to use deep cognitive strategies (Jiwen et al., 2015), which means that learning engagement and academic performance are positively correlated (Schaufeli et al., 2002). On the basis of this analysis, we proposed the following hypotheses.

H3: Learning engagement plays a mediating role in the influence of parent support on the academic performance of secondary vocational students.

H4: Learning engagement plays a mediating role in the influence of teacher support on the academic performance of secondary vocational students.

Students’ learning engagement is also influenced by intrinsic personality factors – including achievement goal orientation – that reflect the motivation and rationale underpinning individuals’ engagement in achievement-related behaviors. Therefore, achievement goal orientation can be used to indicate students’ attitudes toward homework and academic performance. Achievement goal orientations will generate varying degrees of learning engagement (Yunyun et al., 2021) and further influence the effect of parent and teacher support on students’ academic achievement. On the basis of individual concerns regarding academic success and related evaluation criteria, achievement goal orientation usually includes three types. Students who master the goal orientation focus on adaptive behaviors, have more positive attitudes toward learning, pursue understanding and mastery of knowledge, and improve their personal abilities. Students with performance close to their goal orientation seek to prove their abilities, aiming to obtain positive evaluations from important people, such as teachers and parents. Students with performance-avoidance are afraid of failure, wanting to avoid revealing their deficiencies. In accordance with this analysis, we proposed the following hypothesis.

H5: Achievement goal orientation plays a mediating role in the relationship between parent and teacher support and academic performance.

**FIGURE 1:**
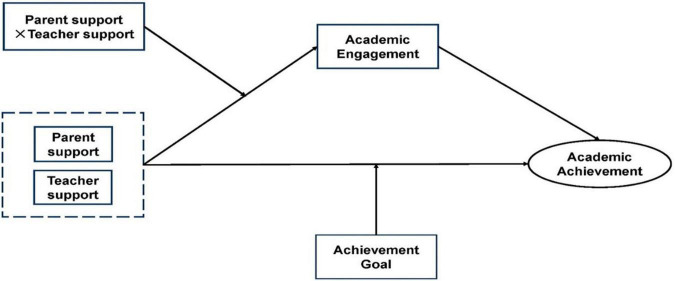
Theoretical model of the influence mechanism of parent and teacher support on secondary school students’ academic performance.

On the basis of H1–H5, we proposed a hypothetical model of the influence mechanism of parent and teacher support on the academic performance of secondary vocational students (Figure 1). Both teacher and parent support impact the development of secondary vocational students’ academic performance, and both have an interactive influence on students’ academic performance. Parent and teacher support affects students’ academic performance through learning engagement, which plays a mediating role. Achievement goal orientation moderates the influence of parent and teacher support on students’ academic performance, and achievement goal orientation plays a moderating role.

## Research Design

### Sample

To uphold ethical guidelines, we informed the study respondents that the questionnaire was for research purposes and their responses would be used anonymously. The responding students participated voluntarily and completed the questionnaire online without their teachers or other school staff present.

This study was conducted in April 2021. The researchers selected four full-time public secondary vocational schools in Shanghai and distributed 200 questionnaires to each school through the Chinese questionnaire platform *wjx.cn* using cluster random sampling. A total of 800 questionnaires were distributed, and 751 questionnaires were collected, with a recovery rate of 93.88%. After excluding 41 invalid questionnaires with a short response time and many missing values, 710 questionnaires were recovered, giving a response rate of 88.75% for valid questionnaires. Of the valid survey sample, 491 students had participated in the public basic course academic proficiency test in Shanghai secondary vocational schools. For the 219 students who had not taken the test, the researchers obtained scores in the form of self-reports. The participants’ demographic information is shown in Table 1.

**TABLE 1:** Demographic characteristics of the sample.

Demographic variables	Category	Frequency (people)	Percentage
Sex	Male	471	66.3%
	Female	239	33.7%
Specialty	Processing and manufacturing	240	33.8%
	Information technology	203	28.6%
	Tourism services	113	15.9%
	Finance, economics and trade	154	21.7%
Grade	First grade	219	30.8%
	Second grade	252	35.5%
	Third grade	239	33.7%
Mother’s education level	Elementary school and below	57	8%
	Junior high school	182	25.6%
	High school	247	34.8%
	Undergraduate	203	28.6%
	Master’s degree and above	21	3%
Father’s education level	Elementary school and below	29	4.1%
	Junior high school	141	19.9%
	High school	317	44.6%
	Undergraduate	202	28.5%
	Master’s degree and above	21	2.9%
Family financial status	High income (monthly income >15,000)	50	7%
	Middle to high income (monthly income 10,000–15,000)	429	60.4%
	Low to middle income (monthly income 2,000–6,000)	210	29.6%
	Low income (monthly income 0–2,000)	21	3%

### Measurements

This study used questionnaires to investigate parent support, teacher support, learning engagement, and achievement goal orientation in secondary vocational education. AMOS (v.23.0, Chicago, IBM, NY, United States) and SPSS (v.25.0, Armonk, NY: IBM Corp., United States) were used to analyze the reliability and validity of the research scale (Table 2). For teacher support, the scale used the *Teacher as Social Contact (TASC) Scale* developed by Wellborn et al. (1988), and revised in 2020 by Rong (2020). The scale was divided into three dimensions and fifteen items. Autonomy support was mainly measured by five items, such as ‘Teachers will encourage me to ask questions about what I have learned.’ Emotion support was measured by five items, such as ‘Teachers are confident in my learning.’ Ability support was measured by five items, such as ‘Teachers will assign exercises with moderate difficulty instead of requiring me to answer questions that are too simple or too difficult.’ The measurement items used a five-point Likert scale, where 1 is ‘very inconsistent’ and 5 is ‘very consistent.’ As the score increased, the teacher support improved. Cronbach’s alpha of the three dimensions was 0.864, 0.889, and 0.876, indicating good reliability of the scale. The latent variable model was established for the three sub-dimensions of the teacher support scale, and the model fit index was good (χ^2^/df = 2.647, CFI = 0.976, TLI = 0.971, RMSEA = 0.048, SRMR = 0.031, GFI and AGFI > 0.9, CR values = 0.866, 0.890, and 0.878, and AVE = 0.564, 0.619, and 0.591). The CR and AVE values of each dimension met the corresponding standards and the factor loading of each item was between 0.715 and 0.897. The scale has good convergent and constructs validity.

**TABLE 2:** Questionnaire reliability indicators.

	Teacher support	Parental support	Academic engagement	Achievement goal orientation
χ^2^	230.315	201.754	196.030	246.498
df	87	74	87	101
χ^2^/df	2.647	2.726	2.253	2.441
CFI	0.976	0.978	0.981	0.980
TLI	0.971	0.972	0.977	0.976
RMSEA	0.048	0.049	0.042	0.045
SRMR	0.031	0.032	0.030	0.045
GFI	0.961	0.964	0.972	0.971
AGFI	0.923	0.933	0.958	0.939
FL	0.715–0.897	0.720–0.881	0.699–0.886	0.676–0.917

The ‘Parent Support’ scale was based on the *Perceived Social Support Scale (PSSS)* (Blumenthal et al., 1987) adapted for Chinese by Wei et al. (2016) and applied in Chinese contexts with good reliability and validity. The tool is divided into 3 dimensions and 14 items. Autonomy support included five items, such as ‘My parents will encourage me to express my opinions.’ Emotion support comprised five items, including ‘My parents always spare time to accompany me.’ Ability support included four items, such as ‘My parents will help me make a good study plan.’ Again, items were evaluated using a five-point Likert scale, where 1 means ‘very inconsistent,’ and 5 means ‘very consistent.’ As the score increased, the level of parent support increased. The Cronbach’s α of the three dimensions was 0.891, 0.859, and 0.869, indicating good reliability. The latent variable model was established for the three sub-dimensions of the scale, and the model fit index was again good (χ^2^/df = 2.726, CFI = 0.978, TLI = 0.972, RMSEA = 0.049, SRMR = 0.032, GFI and AGFI > 0.9, CR values = 0.901, 0.864, and 0.872, AVE values = 0.645, 0.560, and 0.630). The CR and AVE values of each dimension met the corresponding standards, and the factor loading of each item was between 0.720 and 0.881. The research scale thus had good convergent and construct validity.

The Learning Engagement Scale referred to Fredricks et al.’s (2004) research and divided learning engagement into three dimensions of behavior, emotion and cognition. And the scale was revised also on the basis of the eleven items of engagement domain in *The Student Self-Report For Middle School in Research Assessment Package scale* (RAPS-SM) developed by Connell (1998) and *the Chinese Vocational School Student Learning Engagement Scale* developed by Chinese scholar Wei Xiangyun (2019). The learning engagement part consists of 3 measurement dimensions and 15 items. Behavior engagement included five items, such as ‘I will do a good preview before class.’ Emotion engagement comprised five items, such as ‘I am happy to study in this school.’ Cognition engagement included four items, such as ‘Facing the learning pressure, I will self-regulate and relieve the pressure.’ As for the other scales, a five-point Likert scale was used and higher overall scores indicated greater learning engagement. Cronbach’s α coefficients of the subscale and its three dimensions were 0.859, 0.880, and 0.875, and the reliability coefficient was higher than 0.7, indicating good reliability. A latent variable model was established for the three sub-dimensions of the scale, with a good model fit index (χ^2^/df = 2.253, CFI = 0.981, TLI = 0.977, RMSEA = 0.042, SRMR = 0.030, GFI and AGFI > 0.9, CR values = 0.861, 0.884, 0.877, and 0.848, and AVE values = 0.555, 0.605, 0.589, and 0.650). The CR and AVE values of each dimension met the appropriate standards, and the factor loading of each item ranged between 0.699 and 0.886. Thus, this scale also showed good convergent and construct validity.

The ‘Achievement Goal’ scale was translated from Elliot and Chureh’s ‘Achievement Goal Questionnaire’ by Jiang Jingchuan (Jingchuan and Huashan, 2006), and has demonstrated reliability and validity in the Chinese context. This scale includes 3 dimensions and 17 items. Mastery goal included five items, such as ‘Trying to learn as much as possible in class makes more sense than getting a good score.’ The performance approach consisted of seven items, such as ‘It’s important for me to be better than most of my classmates.’ Performance avoidance included five items, such as ‘I often consider that my academic performance cannot lag too far behind.’ The measurement items used a five-point Likert scale, as for the previous scales described. As the overall score obtained increased, the achievement goal orientation became stronger. The test results showed that the Cronbach’s α of the three dimensions was 0.907, 0.900, and 0.891, and reliability was good. A latent variable model was established for the three sub-dimensions of the achievement goal orientation scale, and the model fit index was good (χ^2^/df = 2.441, CFI = 0.980, TLI = 0.976, RMSEA = 0.045, SRMR = 0.045, GFI and AGFI > 0.9, CR values = 0.908, 0.902, and 0.891, AVE values = 0.665, 0.607, and 0.621). The CR and AVE values of each dimension also met the corresponding standards, and the factor loading of each item was between 0.676 and 0.917. The research scale thus has good convergent and construct validity.

The level of academic achievement can reflect the role of parent and teacher support in the growth of secondary vocational students. High school entrance examinations focus mainly on students’ cognitive skills as a selection mechanism. This results in secondary vocational students’ cognitive skills lagging behind those of ordinary high school students. However, the entrance examination does not effectively discern differences in the operational skills of secondary vocational school students and ordinary high school students. Therefore, taking academic achievement as an indicator may better reflect the role of parent and teacher support in the growth of secondary vocational school students. To avoid differences caused by the varying assessment content and standards of schools, grades, and major courses, this study used the results of the Unified Academic Level Examination of Shanghai Secondary Vocational Schools as their academic performance. Each secondary vocational student takes the test in the second grade to get a score, which also qualifies the student to obtain a graduation certificate. The scores are rated into five levels of A, B, C, D, E: A (top 20%), B (20–40%), C (40–60%), D (60–80%), E (bottom 20%). A question was set in the questionnaire to ask whether the respondents have participated in this examination, and then to ask them to choose the corresponding score levels of A, B, C, D, E. While the examination for the first-year students is conducted by each school instead of a unified one of Shanghai secondary vocational schools. We collected data on the academic performance of the first-year students based on the same five levels as the Unified Academic Level Examination: A (top 20%), B (20–40%), C (40–60%), D (60–80%), E (bottom 20%). The schools generally evaluate the students according to their class rankings and each student knows his or her ranking. The researcher set items in the questionnaire, asking the students voluntarily choose their levels according to their class rankings: A (top 20%), B (20–40%), C (40–60%), D (60–80%), E (bottom 20%).

## Results

### The Influence of Parent and Teacher Support on the Academic Performance of Secondary Vocational School Students

**FIGURE 2:**
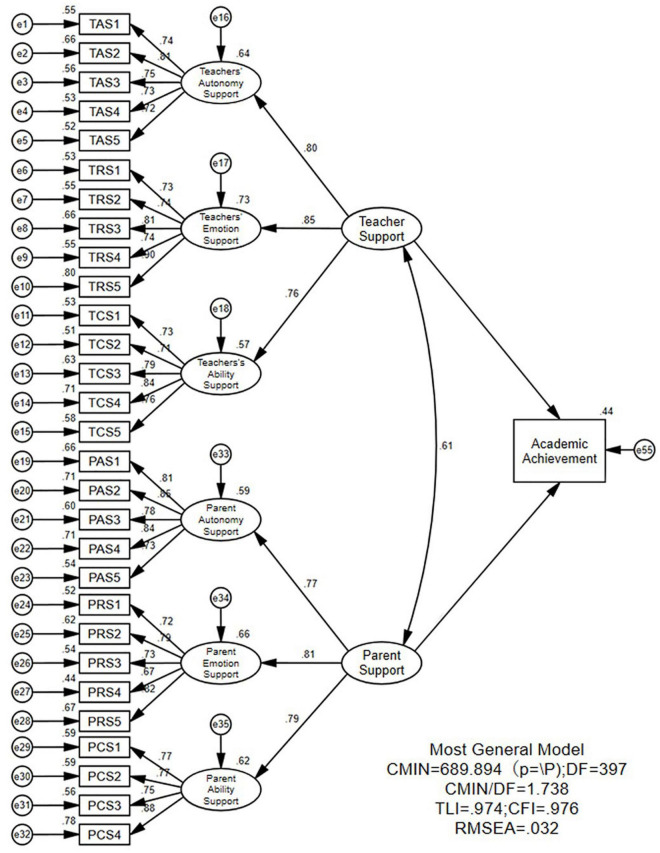
Model of the direct effect of parent and teacher support on academic performance. TAS, TRS, TCS, PAS, PRS, and PCS refer to teacher autonomy support, teacher emotion support, teacher ability support, parent autonomy support, parent emotion support, parent ability support.

Perceived parent and teacher support was examined from three dimensions: autonomy, emotion, and ability support. Parents’ autonomy and emotion support, and teachers’ autonomy, emotion, and ability support were measured by five variables each, while four observed variables measured parents’ ability support. The results were as follows: χ^2^/df = 1.738, CFI = 0.976, TLI = 0.974, and RMSEA = 0.032. Each indicator met the adaptation standard and the model fits well. The standardized factor loadings for each observed variable ranged from 0.67 to 0.88. Standardized path coefficients for teacher support on academic achievement were 0.47 (*p* < 0.001) and for parent support on academic achievement were 0.26 (*p* < 0.001). These results show that both parent and teacher support significantly affect the academic performance of secondary vocational school students, and verify hypothesis H1 (Figure 2).

### The Intermediary Role of Learning Engagement

The structural equation model was used to examine the intermediary role of learning engagement between parent and teacher support and the academic performance of secondary vocational school students. This research first used AMOS 23.0 to establish a latent variable structural equation model with one pair of independent and dependent variables of ‘parent and teacher support’ and ‘academic performance of secondary vocational school student.’ The results showed that the total effect model fits well. Learning engagement was subsequently incorporated into the indirect effects model as a mediating variable. It was found that χ^2^/df= 1.475, CFI = 0.976, TLI = 0.974, and RMSEA = 0.026. Teacher support had a significant effect on learning engagement (β = 0.470, *p* < 0.001), and parent support also exerted a significant effect on learning engagement (β = 0.279, *p* < 0.001). Thus, both independent variables are valid in the first half of the intermediary path. Learning engagement significantly impacted performance (β = 0.426, *p* < 0.001), indicating the second half of the intermediary path was also valid.

On the premise that both intermediary paths are valid, the researchers used a Bootstrapping method to conduct 5,000 simulation analyses and calculate the 95% confidence interval of bias correction for indirect effects. The direct and total effects were also analyzed by the same method. The results (Table 3) show that teacher support had a direct impact on student performance (effect = 0.270, 95% CI = [0.180, 0.355]), and an indirect impact on student performance through learning engagement (effect = 0.200, 95% CI = [0.146, 0.266]). Parental support also exerted a direct effect on students’ performance (effect = 0.139, 95% CI = [0.044, 0.223]), and an indirect effect on performance through learning engagement (effect = 0.119, 95% CI = [0.070, 0.177]).

**TABLE 3:** Bootstrap test for direct, indirect, and total effects (normalized).

Variables	Effect type	Effect value	Bootstrap			Percentage
			SE	95% CI lower bound	95% CI upper bound	
Teacher support	Direct effect	0.270	0.045	0.180	0.355	57.45%
	Indirect effect	0.200	0.030	0.146	0.266	42.55%
	Total effect	0.470	0.042	0.384	0.552	
Parental support	Direct effect	0.139	0.046	0.044	0.223	53.90%
	Indirect effect	0.119	0.027	0.070	0.177	46.10%
	Total effect	0.258	0.049	0.160	0.349	
Effect difference	Direct effect	0.131	0.075	−0.018	0.276	
	Indirect effect	0.081	0.046	−0.008	0.174	
	Total effect	0.213	0.086	0.045	0.383	

*All effect differences are results of teacher support–parent support.*

**FIGURE 3:**
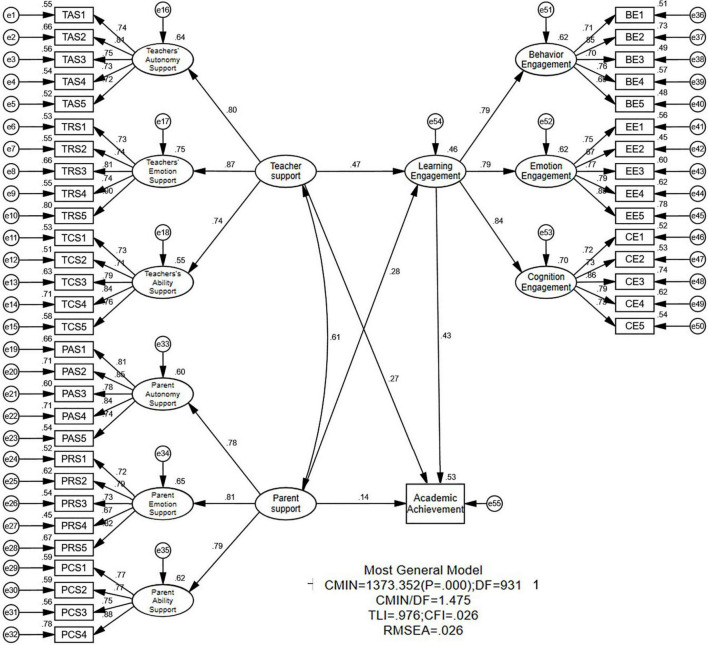
Indirect effects model (normalized). TAS, TRS, TCS, PAS, PRS, PCS, BE, EE, and CE refer to teacher autonomy support, teacher emotion support, teacher ability support, parent autonomy support, parent emotion support, parent ability support, learning engagement, emotional engagement, and cognitive engagement.

Further calculation of the effect difference between teacher support and parent support found no difference in either the direct effect (difference = 0.131, 95% CI = [−0.018, 0.276]) or the indirect effect (difference = 0.081, 95% CI = [−0.008, 0.174]). However, there was a significant difference in the total effect (difference = 0.213, 95% CI = [0.045, 0.383]). These results (Figure 3) show that although there was no significant difference between teacher and parent support in their specific ways of influence, the influence of teacher support on academic performance was greater than that of parent support because of accumulated factors, such as long effective contact time, close interaction and teachers’ influence on students.

In summary, learning engagement plays a partial intermediary role in the influence of parent support on students’ academic performance. It also plays a partial mediating role in the influence of teacher support on students’ academic performance. Hypotheses H3 and H4 are valid (Figure 3).

### The Interaction Between Parent and Teacher Support

As a process variable, learning engagement was influenced by parent and teacher support and had a significant positive impact on students’ academic performance. This study further took teacher and parent support as independent variables and constructed a multiplicative term of them, making a hierarchical regression analysis with learning engagement as the dependent variable. The result (Table 4) showed an interaction between teacher and parent support (β = 0.191, *p* < 0.001).

**TABLE 4:** Regression analysis of the interaction effect of parental and teacher support (normalized).

Variables	M1			M2		
	β	*t*	*p*	β	*t*	*p*
Teacher support	0.357	9.944	0.000	0.356	10.133	0.000
Parent support	0.283	7.897	0.000	0.289	8.229	0.000
Interaction terms				0.191	5.666	0.000
*R* ^2^	0.306			0.336		
*F*	155.997 (*p* < 0.001)			119.276 (*p* < 0.001)		
Δ*R*^2^				0.030		
Δ*F*				32.106 (*p* < 0.001)		

We then used the selected-point method to perform a simple slope test and found that when parent support is low (−1 SD), teacher support has a significant positive effect on learning engagement (β = 0.165, *p* < 0.001). When parent support is high (+1 SD), as the level of teacher support increases, the level of student learning engagement will increase faster (β = 0.547, *p* < 0.001).

**FIGURE 4:**
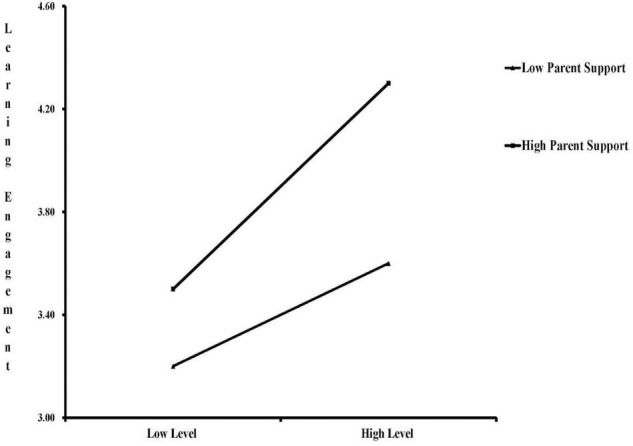
Interaction effect diagram between parent and teacher support.

To sum up, there is an interaction between parent and teacher support on the academic performance of secondary vocational school students, and hypothesis H2 is supported (Figure 4).

### The Moderating Effect of Achievement Goal Orientation

Hierarchical regression analysis was adopted to test the moderating effect of achievement goal orientation. The dependent variable in this study was academic performance, and the independent variables were parent and teacher support. Three moderating variables needed to be tested one by one, namely, mastery goal, performance-approaching goal, and performance-avoiding goal. Thus, the independent variables, the moderated variables, and the product of the independent variable and the moderated variable were incorporated into the regression equation to test the moderating effect. To avoid the problem of variable collinearity, all variables need to be mean-centered before constructing the product term. Then, the centered variables are incorporated in the regression equation to further investigate the moderating effect (Wei and Yingying, 2018).

#### The Moderating Effect of Mastery Goal

A hierarchical regression analysis was conducted with parent and teacher support and mastery goals as the independent variables. The multiplicative term was constructed, and academic achievement was used as the dependent variable. The results are shown in Table 5, where a significant interaction between them (β = 0.177, *p* < 0.001) can be observed.

**TABLE 5:** Results of regression analysis of moderating effects of mastery goal orientation (normalized).

Variables	M1			M2		
	β	*t*	*p*	β	*t*	*p*
Mastery goal orientation	0.068	1.944	0.052	0.089	2.605	0.009
Support	0.406	15.211	0.000	0.390	15.003	0.000
Interaction terms				0.177	6.775	0.000
*R* ^2^	0.279			0.323		
*F*	136.479			112.064		
Δ*R*^2^				0.044		
Δ*F*				45.899		

**FIGURE 5:**
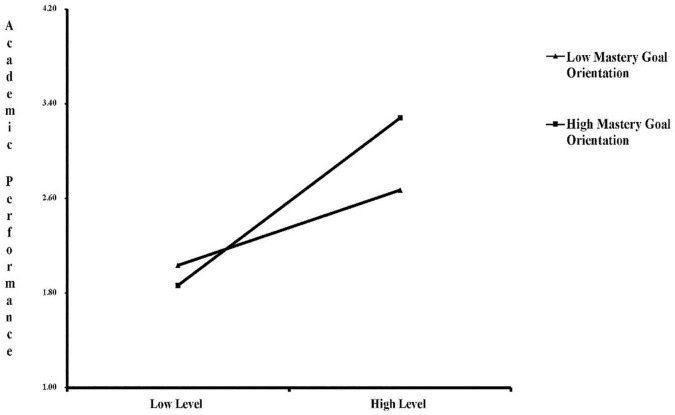
Moderating role of mastery goal orientation in the relationship between parent and teacher support effects on academic performance.

To further understand the moderating effect of mastery goal orientation on the relationship between parent and teacher support and academic achievement, the sample was divided into high mastery goal orientation (+1 SD) and low mastery goal orientation (−1 SD) (Aiken and West, 1991). The moderating effect of mastery goal orientation on the relationship between parent and teacher support and academic performance was drawn according to the simple slope of the effect of parent and teacher support on academic performance. Figure 5 shows that the direction of the influence of parent and teacher support on academic performance does not change with the level of achievement goal orientation. This means that irrespective of an individual’s mastery goal orientation level, academic performance will increase with the level of parent and teacher support, at different growth rates. The positive effect of parent and teacher support on the academic performance of those with high mastery goal orientation (+1 SD) is stronger than for students with low mastery goal orientation (−1 SD). That is, mastery goal orientation strengthens the positive effect of parent and teacher support on the academic performance of students.

#### The Moderating Effect of Performance-Approaching Goal

A hierarchical regression analysis was conducted with parent and teacher support and performance-approaching goal as the independent variables, the multiplicative term, and academic achievement as the dependent variable (Table 6). A significant interaction was found between them (β = 0.151, *p* < 0.001).

**TABLE 6:** Results of regression analysis of moderating effects of achievement-approaching (normalized).

Variables	M1			M2		
	β	*t*	*p*	β	*t*	*p*
Achievement close to target orientation	0.121	3.321	0.001	0.127	3.540	0.000
Support	0.395	14.851	0.000	0.385	14.685	0.000
Interchange items				0.151	5.354	0.000
*R* ^2^	0.286			0.314		
*F*	141.476			107.565		
Δ*R*^2^				0.028		
Δ*F*				28.669		

**FIGURE 6:**
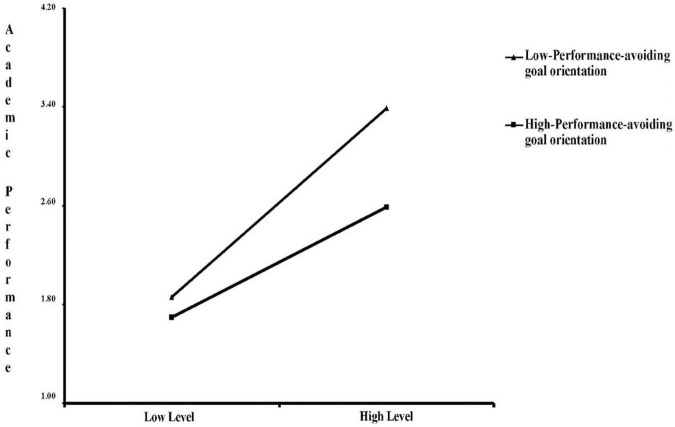
Moderating role of achievement-approaching goal orientation in the relationship between parent and teacher support effects on academic performance.

To further understand the moderating effect of performance-approaching goals on the relationship between parent and teacher support and academic achievement, the sample was divided into high-performance-approaching goal orientation (+1 SD) and low-performance-approaching goal orientation (−1 SD). The simple slope of parent and teacher support on academic performance indicated a moderating effect of performance-approaching goal orientation on the relationship between parent and teacher support and academic performance (Figure 6). The direction of the influence of parent and teacher support on academic performance did not change with the level of performance-approaching goal orientation. Compared with students with low performance-approaching goal orientation (−1 SD), there was a stronger positive effect of parent and teacher support on students’ academic performance with high performance-approaching goal orientation (+1 SD). In other words, performance-approaching goal orientation strengthens the positive effect of parent and teacher support on the academic performance of students.

#### The Moderating Effect of Performance-Avoiding Goal

A hierarchical regression analysis was conducted with parent and teacher support and performance-avoiding goals as the independent variables, the multiplicative term, and academic achievement as the dependent variable (Table 7). The results are shown in Table 6, indicating a significant interaction between them (β = 0.151, *p* < 0.001).

**TABLE 7:** Results of regression analysis of moderating effects of achievement-avoiding goal orientation (normalized).

Variables	M1			M2		
	β	*t*	*p*	β	*t*	*p*
Achievement avoidance goal orientation	0.114	3.155	0.002	0.117	3.312	0.001
Support	0.398	15.027	0.000	0.388	14.882	0.000
Interchange items				0.151	5.354	0.000
*R* ^2^	0.285			0.312		
*F*	140.737			106.821		
Δ*R*^2^				0.027		
Δ*F*				28.170		

**FIGURE 7:**
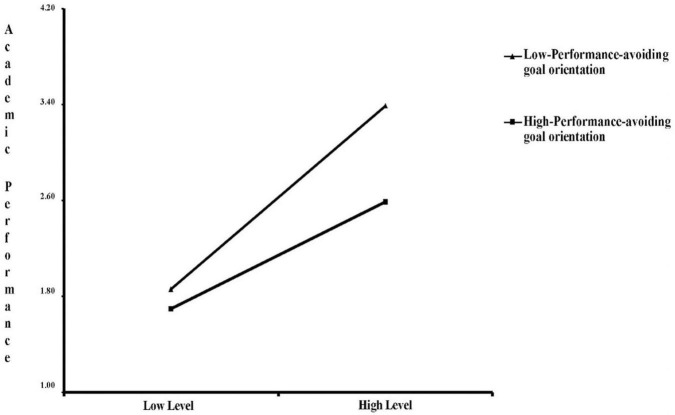
Moderating role of achievement-avoiding goal orientation in the relationship between parent and teacher support effects on academic achievement.

To clearly understand the moderating effect of performance-avoiding goals on the relationship between parent and teacher support and academic achievement, the sample was divided into high-performance-avoiding goal orientation (+1 SD) and low-performance-avoiding goal orientation (−1 SD). The simple slope of parent and teacher support on academic performance indicated a moderating effect of performance-avoiding goal orientation on the relationship between parent and teacher support and academic performance (Figure 7). Regardless of individual performance-avoiding goal orientation, academic performance will increase with parent and teacher support, but at different growth rates. The effect of parent and teacher support on students’ academic performance with low performance-avoiding goal orientation (−1 SD) is stronger than for students with high performance-avoiding goal orientation (+1 SD).

To sum up, achievement goal orientation plays a significant moderating role in the influence of parent and teacher support on the academic performance of secondary vocational students, verifying hypothesis H5.

**FIGURE 8:**
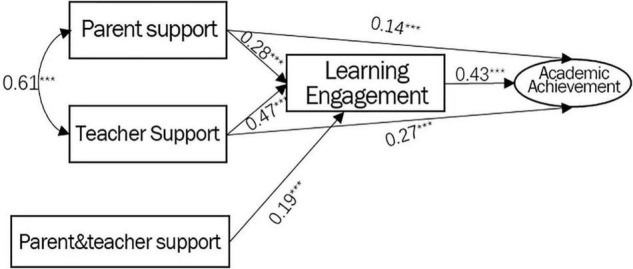
Model diagram of parent and teacher support affecting secondary school students’ academic performance.

The current study investigated the influence mechanism of parent and teacher support on the academic performance of secondary vocational school students, using a theoretical model and empirical data. Parent and teacher support significantly influences secondary vocational school students’ academic performance. Learning engagement plays a partial mediating role, and goal orientation plays a moderating role in the relationship between parent and teacher support and academic performance. Furthermore, there is a significant interaction between parent and teacher support on secondary vocational school students’ learning engagement (Figure 8). These findings play an important guiding role in developing effective measures to improve the academic level of secondary vocational school students – discussed in this section in further detail.

## Discussion

Based on the self-determination theory and social support theory, this study explored the influence mechanism of parent support and teacher support on the academic performance of secondary vocational students. An empirical analysis of the relationship between parent support, teacher support, learning engagement, achievement goal orientation, and academic performance was conducted. The research conclusion showed that parents and teachers, as significant others in the social network of secondary vocational students, can effectively influence their academic performance by providing autonomous support, ability support, and emotional support. Learning engagement, as the amount of physical and psychological energy (Astin, 1984), played a mediating role in the process of parent support and teacher support affecting students’ academic performance. Parent support and teacher support had interactive effects, and the degree of interactive effects affected the variation of learning engagement as well. Students’ achievement goal orientation could moderate the influence of parent support and teacher support on academic performance. And there are differences in the degree of adjustment of different types of achievement goal orientation.

The research results reflected a positive effect of parent support and teacher support on the development of students’ academic performance. It was consistent with the views of scholars (Epstein and Salinas, 2004; Zhe et al., 2019), which confirmed that the social support obtained by the individual will have a positive effect on individual development as stated social support theory (House et al., 1988). This paper further divided the social support from parents and teachers into three categories: autonomous support, ability support, and emotional support (Ryan and Deci, 2000). It was found that all three types of support had significant impacts on students’ academic performance, and the types of support resources were clearly analyzed. The study confirmed that the supports provided by parents and teachers were beneficial to meet the needs of students, which further stimulated their intrinsic motivation to learn, and promoted the development of academic achievement. This was consistent with the viewpoint of the causal path that the environment had an impact on individual behavior, as stated by self-determination theory, and that it played an important role in the motivation and change of individual behavior (Ryan and Deci, 2000). The conclusion of the study confirmed students’ ability to transform external requirements into inner identity value (Ryan and Deci, 2000), and external conditions’ influence on this ability (Ryan, 1995), that is, when the individual faced the inability to arouse the intrinsic motivation, the various supports from the external environment can enable students to perceive a sense of autonomy, ability, and belonging, which led to a better possibility for them to realize the transformation of extrinsic motivation to intrinsic motivation (Ryan, 1995; Vansteenkiste et al., 2006). This study further found that there were differences in the impact of parent and teacher support on the academic performance of secondary vocational students. For these students, both the mastery goal orientation and the achievement approaching goal orientation can strengthen the positive effect of parent and teacher support on academic performance. While the achievement avoidance orientation will weaken the positive effect of parent and teacher support on it. Specifically, we illustrate the research outcomes from the following three aspects:

### Construct a Supportive and Collaborative Family-School Environment to Improve Students’ Learning Engagement

Parents and teachers both have a significant positive impact on the academic performance of secondary vocational school students by providing autonomy, emotion, and ability support. This is consistent with previous studies that showed that parent and teacher support could promote the development of students’ academic performance and their learning engagement (Zong et al., 2017; Wenye et al., 2020; Wei, 2021). However, the two types of support also interact, further suggesting a consistent influence of parent and teacher support on learning engagement. When students perceive a higher level of parent support, together with an increase in teacher support, the level of individual learning engagement will increase faster. In contrast, when they perceive a low level of parent support, even if teacher support is improved, the individual learning engagement level stays low.

Teacher and parent support is an important way of improving students’ academic performance, and their collaborative support can be even more effective. Thus, constructing a supportive and collaborative family-school environment is important to improve secondary vocational school students’ academic performance (Ying and Xi, 2021). The findings also show that environment plays a role in autonomy, emotion, and ability support. First, students need to receive autonomy support to meet their autonomy needs (Qin et al., 2013). Parents and teachers should provide opportunities for choice and decision-making in students’ learning and listen to their ideas (Huiyan and Jiang, 2021). At the same time, secondary vocational students are not yet psychologically mature, although vocational education is employment-oriented and directly targeted at the labor market in contrast with general education (Yunbo, 2021). Secondary vocational education considers the cultivation of professional and technical personnel required by relevant regional industries as its goal. Thus, secondary vocational graduates are exposed to occupational content and the job market earlier than ordinary school graduates (Xiangquan and Hui, 2018; Wuniriqiqige and Yuzhao, 2020) and need appropriate guidance from parents and teachers in the self-determination scenarios.

Second, parents and teachers, as significant people in the growth of secondary vocational students, need to provide support to meet their students’ emotional needs (Wenzhu et al., 2016). They can affirm students’ performance through positive speech and behavior, ensuring students fully perceive love, affirmation, and support, and enhancing the trust between parents and children and teachers and students (Jingjing, 2018). Parents and teachers should emphasize students’ strengths, encouraging and supporting them to participate in vocational skills competitions and other activities to show their abilities and gain confidence in their expertise area (Hong and Jie, 2011).

Secondary vocational education occurs after compulsory education when students are in a new learning stage. Parents and teachers need to treat them differently, pay attention to their emotional needs and pass on positive attitudes (Guoqiu, 2019; Peng, 2021). These students need to feel supported in the family-school environment to meet their ability needs (Huiyan and Jiang, 2021). Teachers should create appropriate assignments based on students’ current and potential cognitive levels, pay attention to individual differences, attach importance to process evaluation, and establish learning files for students at different development levels. Keeping records of the learning assignments completed by students will help them experience a sense of competence and accomplishment (Wiliam, 2011). The ability support provided by parents is limited by their own knowledge levels and ability, in contrast with that of teachers. Thus, it may involve more material and economic level support (Defeng and Simiao, 2021). Parents of secondary vocational school students also have strong expectations of their children. They need to sensitively consider the psychological state of their children on entering secondary vocational schools to avoid psychological pressure in the family environment caused by unsatisfactory academic performance (Xue and Yan, 2021). Parents should pay attention to their children’s psychological needs and understand their learning problems through timely communication with teachers (Wenzhu et al., 2016). They can also help their children set feasible learning goals (Wenzhu et al., 2016). Parents need to manage their own expectations, avoiding unrealistic expectations that lead to frustration with their child – or expectations that are too low and lead to unfulfilled potential and loss of learning motivation. Secondary vocational schools can guide parents to support students’ academic development through training, teacher guidance, and other methods (Jiali and Yongmei, 2021).

### Improve the Level of Learning Engagement to Promote Students’ Academic Development

Learning engagement plays a partial mediating role in the effect of parent and teacher support on students’ academic performance. This means that parent and teacher support can influence students’ academic performance by affecting their learning engagement. First, self-determination theory holds that students’ learning engagement is closely related to the external environment (Vansteenkiste et al., 2006). As the parent and teacher support increases, the impact on learning engagement will become more positive. Second, results show that the time and energy invested by students in learning activities are important variables that predict their academic development, verifying that environmental factors, such as parent and teacher support, can affect the learning process and academic performance. Finally, we found no significant difference in the direct and indirect effect of the interaction effect between teacher and parent support. However, there was a significant difference in the total effect. This shows that the influence of teacher support on the academic performance of secondary vocational school students is greater than that of parent support. This may be because students spend more time in schools, and teachers’ influence is dominant in their education (Wei, 2021). Secondary vocational students are adolescents, eager to develop their independence and self-management skills and reduce parental influence (Qin et al., 2013). Therefore, the role of teacher support is more significant than parent support.

We suggest that targeted measures be taken to improve the learning engagement of secondary vocational students. First, it is necessary to reinforce the student-centered quality concept, establish a complete educational support system, and attach importance to students’ learning process and experience (Hao and Fan, 2021). Second, teachers’ cultural knowledge and their ability to design technical skills courses should be improved through innovative teaching methods and ensure that they stimulate students’ learning enthusiasm (Shengqing, 2021). Teachers should help students become independent, provide sufficient learning resources, meet the unique learning interests and needs of secondary vocational students, improve learning engagement, and cultivate students’ ability to become active participants in learning. Finally, the dynamic nature of students’ learning should be carefully monitored (Wenzhu et al., 2016). Measurement tools should be developed and applied to understand students’ learning engagement. Such tools will help provide teachers and parents with an objective view of changes in learning engagement, enabling them to offer timely academic guidance (Yan, 2021).

### Establish a Positive Learning Concept to Stimulate Students’ Mastery Goal Orientation

Achievement goal orientation can moderate the influence of parent and teacher support on academic performance (Wei, 2021). The study revealed changes in the academic performance of students with different types of achievement goal orientations under different levels of support from parents and teachers. First, on the basis of achievement goal theory, this study analyzed the moderating effects of three types of achievement goal orientations on the relationship between parent and teacher support and academic performance. Mastery goal orientation has a significant moderating effect on the impact of parent and teacher support on secondary vocational school students’ academic performance. Parent and teacher support has a stronger positive impact on students with high mastery goal orientation than students with low mastery goal orientation. The performance-approaching goal orientation has a significant moderating effect on the influence of parent and teacher support on the academic performance of secondary vocational school students. Teachers’ emotion support has a stronger positive effect on the academic performance of students with low performance-avoidance goal orientation than students with higher performance-avoidance goal orientation. In other words, performance-avoiding goal orientation weakens the positive influence of parent and teacher support on academic performance.

Second, the study found that students with high mastery goals depend more on the external environment in the learning process and are more vulnerable to the influence of parent and teacher support than students with low mastery goals. Self-determination theory suggests that students need to satisfy their basic psychological needs such as ability, emotion, and autonomy support, and are eager to acquire knowledge and improve their ability (Wenzhu et al., 2016). However, at this stage of their compulsory education, they may be frustrated with their learning and their basic needs may not have been effectively met (Zhu et al., 2017). Typically, this is because of inadequate mastery of learning engagement, learning methods and strategies, and lack of support from parents and teachers. Secondary vocational students need to gain recognition and respect from others and demonstrate their abilities to attract more support from parents and teachers (Wenzhu et al., 2016). This also explains the phenomenon seen in this study where students with high mastery goal orientation improved significantly with more support from parents and teachers.

Parents and teachers should take effective measures to create an environment suitable for the needs of secondary vocational students and gradually guide them to pursue mastery goal orientation (Jingchuan and Huashan, 2006). Parents and teachers need to pay more attention to students’ ability acquisition, and focus on their development, guiding them to awareness of the significance of learning, the value of effort, and goal-oriented learning motivation (Xuji et al., 2020; Jialin et al., 2021; Ningning et al., 2021). Ultimately, secondary vocational school students need an environment of appreciation and encouragement that attaches importance to their overall development and helps them rebuild their confidence in learning and establish an appropriate view of learning.

## Conclusion

### Implication

Based on the self-determination theory, this study constructs an influence mechanism model of teacher support and parent support on the academic performance of secondary vocational students and verifies the internal variables of the mechanism model through empirical research. Compared with the previous studies, this study newly finds that parent support and teacher support have an interactive effect on the academic performance of secondary vocational students, and explores the principle of this effect on students’ academic performance through learning engagement. In exploring the moderating effect of achievement goal orientation, we further find that different types of achievement goals of secondary vocational students will have a differential impact on their academic performance. The students with high mastery goal orientation are more susceptible to the external environment and are more sensitive to the perception of parent and teacher support. This study revealed the mechanisms and principles that affect the academic performance of secondary vocational students from the psychological perspective and further revealed the “black box” of secondary vocational students’ academic development. In addition, this research provided effective support for guiding the students’ cultivation of future secondary vocational students, stimulating their learning motivation, training scientific spirit, and enhancing their ability to participate in society.

### Limitations and Future Study

Due to the limitation of research time, research resources, and the influence of COVID-19, this research still has potential for further research. Firstly, the samples were only collected from Shanghai. Researchers can conduct data collection in a wider range in the future to verify the applicability of this mechanism. Secondly, because the academic achievement improvement of secondary vocational students is strongly influenced by cultural contexts, researchers can also further compare the differences in the influence mechanism in different regions and countries. Thirdly, based on the social-support theory, this study only selected the resources provided in the two types of places of school and family, that is, the influence of teacher support and parent support on students’ academic performance. It’s recommended that researchers can examine more research variables, such as peer support, community support, etc., that affect the academic performance of secondary vocational students. Fourthly, since secondary vocational students are not yet mentally mature and their learning ability is still developing, researchers can conduct further research to explore the dynamic changes in the impact of parent support and teacher support on the academic performance of secondary vocational students.

## Data Availability Statement

The original contributions presented in the study are included in the article/supplementary material, further inquiries can be directed to the corresponding author.

## Ethics Statement

Ethical review and approval was not required for the study on human participants in accordance with the local legislation and institutional requirements. Written informed consent to participate in this study was provided by the participants. Written informed consent was obtained from the individual(s) AND/OR minor(s)’ legal guardian/next of kin for the publication of any potentially identifiable images or data included in this article.

## Author Contributions

XP participated in conceptualization, supervision, the design of experimental methods, practical investigation analysis of experimental data, visualization of experimental results, and the revising of the manuscript. XS participated in the actual investigation, experimental data analysis, experimental results visualization, and the writing of the first draft of the article. ZH participated in the supervision and was also responsible for the specific communication, manuscript revision, submission, and publication. All authors contributed to the article and approved the submitted version.

## Conflict of Interest

The authors declare that the research was conducted in the absence of any commercial or financial relationships that could be construed as a potential conflict of interest.

## Publisher’s Note

All claims expressed in this article are solely those of the authors and do not necessarily represent those of their affiliated organizations, or those of the publisher, the editors and the reviewers. Any product that may be evaluated in this article, or claim that may be made by its manufacturer, is not guaranteed or endorsed by the publisher.
